# A comprehensive overview and qualitative analysis of government-led nutrition policies in Australian institutions

**DOI:** 10.1186/s12889-020-09160-z

**Published:** 2020-06-30

**Authors:** Emalie Rosewarne, Annet C. Hoek, Gary Sacks, Luke Wolfenden, Jason Wu, Jenny Reimers, Kirstan Corben, Michael Moore, Cliona Ni Mhurchu, Jacqui Webster

**Affiliations:** 1 The George Institute for Global Health, The University of New South Wales, Sydney, NSW 2052 Australia; 2 Deakin University, Melbourne, Victoria 3125 Australia; 3 University of Newcastle, Callaghan, NSW 2308 Australia; 4 Victorian Health Promotion Foundation, 15-31 Pelham Street, Melbourne, Victoria 3053 Australia; 5 The University of Auckland, Auckland, 1142 New Zealand

**Keywords:** Food policy, Nutrition standards, Institutions, Qualitative research, Australia

## Abstract

**Background:**

Institutions are a recommended setting for dietary interventions and nutrition policies as these provide an opportunity to improve health by creating healthy food environments. In Australia, state and territory governments encourage or mandate institutions in their jurisdiction to adopt nutrition policies. However, no work has analysed the policy design across settings and jurisdictions. This study aimed to compare the design and components of government-led institutional nutrition policies between Australian states and territories, determine gaps in existing policies, and assess the potential for developing stronger, more comprehensive policies.

**Methods:**

Government-led institutional nutrition policies, in schools, workplaces, health facilities and other public settings, were identified by searching health and education department websites for each Australian state and territory government. This was supplemented by data from other relevant stakeholder websites and from the Food Policy Index Australia website. A framework for monitoring and evaluating nutrition policies in publicly-funded institutions was used to extract data and a qualitative analysis of the design and content of institutional nutrition policies was performed. Comparative analyses between the jurisdictions and institution types were conducted, and policies were assessed for comprehensiveness.

**Results:**

Twenty-seven institutional nutrition policies were identified across eight states and territories in Australia. Most policies in health facilities and public schools were mandatory, though most workplace policies were voluntary. Twenty-four included nutrient criteria, and 22 included guidelines for catering/fundraising/advertising. While most included implementation guides or tools and additional supporting resources, less than half included tools/timelines for monitoring and evaluation. The policy design, components and nutrient criteria varied between jurisdictions and institution types, though all were based on the Australian Dietary Guidelines.

**Conclusions:**

Nutrition policies in institutions present an opportunity to create healthy eating environments and improve population health in Australia. However, the design of these policies, including lack of key components such as accountability mechanisms, and jurisdictional differences, may be a barrier to implementation and prevent the policies having their intended impact.

## Background

In Australia, dietary risks are the second leading mortality risk factor, and responsible for an estimated 14.8% of total deaths [1]. Key dietary risk factors include low intakes of wholegrains, fruit, nuts and seeds, vegetables, fibre, omega-3 fatty acids, polyunsaturated fatty acids and legumes, and excessive sodium consumption, which are all estimated to be among the top 20 mortality risk factors [1]. Many strategies have been initiated, both in Australia and globally, to address the disease burden attributable to dietary risks. In Australia, these strategies include government-led voluntary nutrient reformulation targets [2, 3], education and awareness raising campaigns (e.g. [4]), interpretive front-of-pack labelling [5], and institutional nutrition policies (e.g. [6]).

Institutions, including schools, workplaces and health facilities, are a recommended setting for dietary interventions [7, 8], as they provide access to a large proportion of the population for a prolonged period of time [9, 10] and have existing infrastructure to deliver interventions. One strategy to influence dietary intake, and achieve improvements in population health, is by setting standards or guidelines on the foods and drinks an institution can procure, prepare, provide and sell. Such nutrition policies are particularly important in public institutions, where governments have the ability to define these standards or guidelines, and encourage or mandate adoption of the policies, and can set an example for creating healthy eating environments [7, 8]. Previous studies suggest that these nutrition policies and guidelines, can be effective in increasing availability and purchases of healthy foods in many environments, including institutions [11]. However, the evidence is limited to high-income countries and few interventions have been subject to rigorous evaluations [11]. In addition, compared to the number of known institutional nutrition policies in existence [12], a relatively small proportion have been evaluated, highlighting a key area for future work.

Institutional nutrition policies and guidelines can be implemented at various levels of government, including national, state/provincial or local governments, depending on each level’s jurisdiction [13]. In Australian institutions, nutrition policies and guidelines exist at multiple levels of government. There are some voluntary national policies and guidelines (e.g. [6])*,* however regulations pertaining to many institutions, for example schools and hospitals, are the responsibility of the state and territory governments [14]. As such, many jurisdictions have developed their own policies (e.g. [15, 16]), and state/territory governments encourage or mandate institutions in their jurisdiction to adopt these policies.

It is important to understand whether these state and territory level institutional nutrition policies are consistent with evidence based recommendations (e.g. [13]), to what extent they are being implemented, and the effect of the policies on the food environment, dietary intake or downstream health outcomes. One of the challenges is that the available evidence is limited to particular institutions and settings. The majority of previous research on government-led institutional nutrition policies in Australia has centred on schools [17], with minimal research relating to health facilities and none relating to workplace nutrition policies. Previous work regarding schools has included investigations into compliance with policies and stakeholder perceptions [18, 19], an overview of differences in policy design and implementation across states and territories [20], and a few randomised control trials in the Hunter region of New South Wales [21–23]. However, since these studies were conducted, the Australian Capital Territory [15], New South Wales [24], Northern Territory [25] and Western Australia [26] policies have been revised, and an update of this research would therefore be warranted. In contrast, little research has been conducted on nutrition policies in health facilities. Studies have been limited to assessing compliance in a small number of health facilities, through audits of foods and drinks available [27, 28].

It is evident that the focus of past research was on policy compliance, yet no work has analysed the policy design, including the extent to which they are consistent with evidence-based recommendations (e.g. [13]), or assessed the potential for developing more comprehensive nutrition policies for Australian institutions. Therefore, the aim of this study was to compare the design and components of government-led institutional nutrition policies between Australian states and territories, determine gaps in existing policies, and assess the potential for developing stronger, more comprehensive policies. Implementation of the policies, and the impact of the policies on total dietary intake or downstream health outcomes is also yet to be assessed, however this is outside the scope of the current paper.

## Methods

Health and education department websites for each state and territory government were searched to identify current government-led nutrition policies relating to institutions, including schools, workplaces, health facilities, or other public settings. From the state and territory government websites, other relevant stakeholder websites, including federal government and quasi-government, were identified and searched. Information from the Food Policy Index Australia website, a website that summarises the progress made by Australian governments in line with implementing globally recommended policies, was used to supplement these data [29].

### Inclusion and exclusion criteria

For this study, nutrition policies were defined as policies, guidelines or frameworks pertaining to creating healthy eating and drinking environments within the institution (i.e. increasing the availability and purchases of healthy foods [11, 13]), such as through nutrition standards. Policies for the provision of food to institutionalised individuals (e.g. hospital in-patients) were excluded as these do not aim to improve the food environment, and food for these populations is wholly provided by the government, requiring 100 % of daily nutrient needs to be met.

### Data extraction and analysis

To compare the design and components of government-led institutional nutrition policies, determine gaps in existing policies, and assess the potential for developing stronger, more comprehensive policies, three levels of comparative policy analysis were undertaken: (1) Policy design and components, (2) nutrition standards, and (3) nutrient criteria. Relevant data were extracted from the policy documents by one author (E.R.) and entered into a purpose-built Microsoft Excel spreadsheet based on L’Abbé et al.’s framework for monitoring and evaluating nutrition standards in publicly-funded institutions [13]. For the comparative policy analysis, institution type, policy name and year introduced/revised were extracted, as well as policy design elements such as approach (voluntary/mandatory), components of the policy (nutrition standards, voluntary policy template, implementation guide/tools, guide for catering/fundraising/advertising, extra resources to support implementation, monitoring/evaluation guide/tools, accreditation program), outline of assigned roles and responsibilities, and outline of monitoring and evaluation plan (including qualitative and/or quantitative approach and time points). Data extracted relating to nutrition standards included: policy name, nutrient profiling system used (e.g. traffic light labelling/Health Star Rating (HSR, [5])), and frequency of specific foods permitted in food outlets/canteens and for catering, fundraising and advertising. Information on nutrient criteria was extracted from the nutrition standards documents and compared between jurisdictions for schools, workplaces and health facilities. Nutrient criteria included guidelines or thresholds relating to energy, saturated fat, sodium, sugar and fibre contents (per 100 g and per serve), portion size criteria and Health Star Rating cut-offs where applicable [5].

## Results

### Comparison of policy design and components

Twenty-seven government-led state and territory level institutional nutrition policies were identified across all eight jurisdictions, including seven policies relating to health facilities, eight school nutrition policies, eight workplace policies, and four policies in other settings. Policies varied between states/territories and between types of institutions. Most policies in health facilities and public schools were mandatory, as was the Australian Capital Territory public sector policy, which applies to all public sector workplaces and facilities. All other workplace policies and all policies in other settings were voluntary, as was the Tasmanian schools policy and Victorian health facilities policies. The components of the policies also varied. Of the 27 policies, 24 included nutrition standards (of which 16 were mandatory and eight were voluntary), 22 included guidelines for catering/fundraising/advertising, 21 included implementation guides or tools, 12 included monitoring/evaluation resources, eight provided a voluntary policy template for modification, seven had an accreditation program available and 25 included additional supporting resources. Six out of seven health facility policies and six out of eight school policies outlined roles and responsibilities for implementation and monitoring, though only one workplace policy included this. The inclusion of plans for ongoing monitoring and evaluation was variable, with four out of seven health facility policies and two out of eight school policies specifying evaluation/review timelines, and two out of eight workplace policies recommending timelines. Four workplace and three school policies had accreditation programs. No state or territory had an accreditation program explicitly for health facility policies, however Victorian health services are encouraged to use the workplace program (Table 1).

**Table 1:** Comparison of the design and components of state and territory nutrition policies in Australian institutions

**State/territory and year introduced or most recently updated**	**Name of policy/guideline**	**Approach**	**Components**	**Outline of roles/ responsibilities**	**Outline of monitoring and evaluation plan**
**Health facilities**					
Australian Capital Territory; 2018	Healthy Food and Drink Choices Policy [16, 30–32]	Mandatory	NS, G, R, M/E	Yes	12 months, 24 months only (qualitative and quantitative)
New South Wales; 2017	Healthy Food and Drink in NSW Health Facilities for Staff and Visitors Framework [33, 34]	Mandatory	NS, I, G, M/E	Yes	Annual (quantitative)
Northern Territory; 2017	Healthy Choices Made Easy - Healthy Food and Drink Options for Staff, Volunteers and Visitors in NT Heath Facilities Policy [35–40]	Mandatory	NS, G, R	No	Baseline, 6 months, 18 months only (qualitative and quantitative)
Queensland; 2015 (currently being revised)	A Better Choice. Healthy Food and Drink Supply [41–45]	Mandatory	NS, I, G	Yes	No
South Australia; 2011 (review planned)	Healthy Food and Drink Choices for Staff and Visitors in SA Health Facilities [46–53]	Mandatory	NS, I, G, R	Yes	No
Tasmania	Nil	N/A	N/A	N/A	N/A
Victoria; 2016	Healthy choices: policy guidelines for hospitals and health services [54, 55]	Voluntary	NS, I, G, R, M/E	Yes	6 months, then annually (qualitative and quantitative)
Western Australia; 2017	The Healthy Options WA: Food and Nutrition Policy for WA Health Services and Facilities [56–61]	Mandatory	NS, I, G, R, M/E	Yes	No
**Schools**					
Australian Capital Territory; 2017	ACT Public School Food and Drink Policy [15, 62–70] / National Healthy School Canteen Guidelines [6]	Mandatory for public schools, voluntary for independent schools	NS, I, G, R	Yes	Annual. Full review to be conducted within a 3 year period (date not specified)
New South Wales; 2018	Nutrition in Schools Policy [24, 71–75]	Mandatory for public schools, voluntary for independent schools	NS, I, G, R, M/E	Yes	Every 3 years (date not specified)
Northern Territory; 2017	School Nutrition and Healthy Eating Policy [25, 76–84] / National Healthy School Canteen Guidelines [6]	Mandatory for public schools	NS, G, R	Yes	No
Queensland; 2016	Smart Choices. Healthy food and drink supply strategy for Queensland schools [85, 86]	Mandatory for public schools	NS, I, G, R, M/E	No	No
South Australia; 2015 (review planned)	Right Bite, Healthy Food and Drink Supply Strategy for South Australian Schools and Preschools [87–90]	Mandatory for public schools, voluntary for independent schools	NS, I, R, M/E	Yes	No
Tasmania; 2014	Tasmania School Canteen Handbook. A Whole School Approach to Healthy Eating [91]/ National Healthy School Canteen Guidelines [6]	Voluntary	NS, I, G, R, M/E, AP	No	No
Victoria; 2006	Healthy Canteen Kit [92–95]	Mandatory for public schools	NS, I, G, R, AP	Yes	No
Western Australia; 2017	Healthy Food and Drink Policy [26, 96–99]	Mandatory for public schools	NS, I, G, R, AP	Yes	No
**Workplaces**					
Australian Capital Territory; 2015	Healthier work [100–107]	Voluntary	VT, I, R, M/E, AP	Recommended	Recommended: 6 months, then annually (qualitative)
New South Wales; 2017	Get Healthy At Work [108–111]	Voluntary	NS, G, R	No	No
Northern Territory; 2017	The Healthy Workplace Toolkit [112]	Voluntary	NS, VT, G, R, M/E	No	No
Queensland; 2013	Healthier. Happier. Workplaces [113–117]	Voluntary	VT, I, R, M/E, AP	No	No
South Australia; 2014	A Workplace Health and Wellbeing Toolkit: Step by Step Guide to Developing a Successful Workplace Program [118]	Voluntary	VT, I, R, M/E	No	No
Tasmania; 2013	Healthy Workplace Nutrition Guidelines [119–121]	Voluntary	NS, VT, I, G, R	No	No
Victoria; 2016	Healthy choices: healthy eating policy and catering guide for workplaces [54, 122]	Voluntary	NS, VT, I, G, R, AP	Yes	Recommended: 6 months, then annually (qualitative)
Western Australia; 2017	Healthier Workplace WA [123–132]	Voluntary	NS, VT, I, G, R, M/E, AP	No	Nil
**Other settings**					
Victoria; 2016	Healthy choices: policy guidelines for parks [54, 133]	Voluntary	NS, I, G, R	No	Recommended: 12 month review only (qualitative)
Australian Capital Territory; 2016	ACT Public Sector: Healthy Food and Drink Choices Policy and Vending Machine Management Policy [31, 32, 134, 135]	Mandatory	NS, G, R	Yes	Nil
Queensland: 2014	Choose Well, Live Well - Remote Area Camp Food [136]	Voluntary	NS, I, R, E	No	Nil
Victoria; 2016	Healthy choices: policy guidelines for sport and recreation centres [54, 137]	Voluntary	NS, VT, I, G, R,	Yes	Recommended: 6 months, then annually (qualitative)

*ACT* Australian Capital Territory, *NSW* New South Wales, *NT* Northern Territory, *SA* South Australia, *WA* Western Australia, *NS* Nutrition standards, *VT* Voluntary policy template, *I* Implementation guide/tools, *G* Guide for catering/fundraising/advertising, *R* Extra resources to support implementation, *M/E* Monitoring/Evaluation guide/tools, *AP* Accreditation program

### Comparison of nutrition standards

Most schools and health facilities with nutrition policies had a mandatory nutrition standards, as did the Australian Capital Territory public sector policy. Four state/territory workplace policies, three policies in other settings, one school and one health facility policy included voluntary nutrition standards. All nutrition standards were based on the Australian Dietary Guidelines [138] classification of core and discretionary foods, however two different systems were used to classify the foods. Except for New South Wales, all other states and territories utilised a traffic light labelling system (Table 2). Traffic light classifications were specified as: Red (described as ‘not recommended’, ‘not on the menu’/‘off the menu’, ‘occasional’ foods or foods to ‘avoid’/‘limit’), amber (‘select carefully’/‘choose carefully’) and green (described as the ‘best choice’, ‘always on the menu’/‘fill the menu’, ‘have plenty’, or ‘everyday’ foods). However, the state of New South Wales used the HSR system and portion size criteria to classify foods into ‘everyday’ and ‘occasional’ foods, where ‘everyday’ foods were described as foods from the five food groups and ‘occasional’ foods as foods with less nutritional value than everyday foods [24].

**Table 2:** Comparison of the design of state and territory level procurement policies in Australian institutions

**Jurisdictions and year introduced or updated**	**Name of Procurement Policy/Nutrient Standards**	**System**	**Percentages or frequency of different foods permitted for food outlets/ canteens and vending**	**Percentages or frequency of different foods permitted for catering, fundraising and advertising**
**Health Facilities**				
Australian Capital Territory; 2013	Healthy Food and Drink Choices Policy	TL	**Food Outlets** - Green: Majority, Green and Amber: ≥ 80%, Red: < 20%. **Vending** - Red: < 20%.	**Catering** - Green: Majority. Red: Nil. **Fundraising** - Green: Majority. Red: Nil. **Advertising** - Green: Only.
New South Wales; 2017	Healthy Food and Drink in NSW Health Facilities for Staff and Visitors Framework	HSR	**Food Outlets** and **Vending** - Everyday: ≥75%, Occasional: < 25%. Ban on sugary drinks.	**Catering** - Everyday: ≥75%, Occasional: < 25%. **Advertising** - Everyday: only.
Northern Territory; 2017	Healthy Food and Drink options for Staff, Volunteers and Visitors in Northern Territory Health Facilities: Healthy Choices Made Easy Foods and Drinks Guide	TL	**Food Outlets** and **Vending** - Green and Amber: ≥80% (Aim for ≥50% of green). Red: < 20%.	**Catering** - Red: Nil. **Fundraising** - Red: Nil. **Advertising** - Green: Only.
Queensland; 2007	A Better Choice: Healthy Food and Drink Supply Strategy for QLD Health Facilities	TL	**Food Outlets** - Green and Amber: ≥ 80%. Red: < 20%. Over time increase to ≥50% green choices. **Vending** - Red: Nil.	**Catering** - Red: Nil. **Fundraising** - Red: Nil. **Advertising** - Green: Only.
South Australia; 2011	Healthy Food and Drink Choices for Staff and Visitors in SA Health Facilities Policy	TL	**Food Outlets** and **Vending** - Red: < 20%	**Catering** - Red: Nil. **Fundraising** - Red: Nil. **Advertising** - Red: Nil.
Tasmania	Nil	Nil	Nil	Nil
Victoria; 2016	Healthy Choices: Food and Drink Classification Guide	TL	**Food Outlets** and **Vending** - Green: ≥ 50%, Red: < 20%.	**Catering** - Green: Majority, Amber: Small quantities only. Red: Nil. **Fundraising** - Red: Nil. **Advertising** - Green: Majority, Red: Nil.
Western Australia; 2017	The Healthy Options WA: Making Healthy Food Choices Easier Implementation Guide	TL	**Food Outlets** and **Vending** - Green: ≥ 50%, Red: < 20%.	**Catering** - Green: ≥ 50%, Red: < 20%. **Fundraising** - Green: ≥ 50%, Red: < 20%. **Advertising** - Green: Only.
**Schools**				
Australian Capital Territory; 2015	National Healthy School Canteen Guidelines	TL	**Food Outlets** - Green: Majority, Red: Nil. Ban on sugary drinks. **Vending**: Nil permitted.	**Fundraising**: ≤ two occasions per term.
New South Wales; 2017	The NSW Healthy School Canteen Strategy. Food and Drink Criteria	HSR	**Food Outlets** and **Vending** - Everyday: ≥75%, Occasional: ≤25%. Ban on sugary drinks.	**Advertising** - Everyday: Only.
Northern Territory; 2017	National Healthy School Canteen Guidelines	TL	**Food Outlets** and **Vending** - Green: Majority, Red: Nil.	**Catering** - Green: Majority, Red: Nil. **Fundraising** - Green: Majority, Red: Nil. **Advertising** - Green: Only.
Queensland; 2016 revised	Smart Choices. Healthy Food and Drink Supply Strategy for Queensland Schools.	TL	**Food Outlets** and **Vending** - Red: ≤ two occasions per term.	**Fundraising** - Red: ≤ two occasions per term. **Advertising** - Green: Only.
South Australia; 2004	Right Bite. Healthy Food and Drink Supply Strategy for South Australian Schools and Preschools	TL	**Food Outlets** and **Vending** - Green: Majority, Red: Nil.	**Catering** - Red: ≤ two occasions per term. **Advertising** - Green: Only.
Tasmania; 2014	National Healthy School Canteen Guidelines (2014)	TL	**Food Outlets** and **Vending** - Green: Majority, Red: Nil.	**Catering** - Green: Majority, Red: Nil. **Fundraising** - Green: Majority, Red: Nil. **Advertising** - Green: Only.
Victoria; 2006 (currently being revised)	Healthy Canteen Kit. Food Planner	TL	**Food Outlets** - Green: Majority, Red: Nil. **Vending** - Red: Nil. Ban on sugary drinks and confectionary.	**Catering** - Green: Majority, Red: ≤ two occasions per term. **Advertising** - Green: Majority, Amber: Minority.
Western Australia; 2017	Federation of Canteens in Schools (FOCiS) Food Industry Handbook	TL	**Food Outlets** - Green: ≥ 60%. Amber: < 40%. Red: Nil.	**Catering** - Red: Nil. **Fundraising** - Red: Nil.
**Workplaces**				
Australian Capital Territory; 2015	Nil	Nil	Nil	Nil
New South Wales; 2017	Get Healthy at Work. Healthier Food and Drinks Guide	HSR	**Food Outlets** and **Vending** - Everyday: ≥75%, Occasional: ≤25%.	**Catering** - Everyday: ≥75%, Occasional: ≤25%.
Northern Territory; 2017	The Healthy Workplace Toolkit: Healthy Workplace Nutrition Guidelines	TL	**Food Outlets** and **Vending** Recommendation - Green: ≥50%, Red: < 20%).	**Catering** - Red: Limited. **Fundraising** - Red: Limited.
Queensland; 2013	Nil	Nil	Nil	Nil
South Australia; 2014	Nil	Nil	Nil	Nil
Tasmania; 2013	Healthy Workplace Nutrition Guidelines	TL	Nil	Nil
Victoria; 2016	Healthy Choices: Food and Drink Classification Guide	TL	**Vending** - Red: ≤ 20%.	**Catering** - Green: Majority, Red: Nil. **Fundraising** - Red: Nil for children. **Advertising** - Green: Majority, Red: Nil.
Western Australia; 2017	Healthy Choices: Healthy Futures Healthier Food and Drinks Guide	Nil	**Food Outlets** and **Vending** recommendations - Green: ≥50%, Red: < 20%.	**Catering** recommendations - Green: ≥50%, Red: < 20%.
**Other settings**				
Victoria; 2016	Healthy Choices: Food and Drink Classification Guide	TL	**Food Outlets** and **Vending** - Green: ≥50%, Red: < 20%.	**Catering** - Green: Majority, Red: Nil. **Fundraising** - Red: Nil for children and youth. **Advertising** - Green: Majority, Red: Nil.
Australian Capital Territory; 2016	Healthy Food and Drink Choices Policy - Public Sector	TL	**Food Outlets** - Green: Majority, Green and Amber: ≥ 80%.	**Catering** - Green: Majority, Red: Nil. **Fundraising** - Green: Majority, Red: Nil. **Advertising** - Green: Only.
Queensland: 2014	Choose Well, Live Well - Remote Area Camp Food	TL	Nil	**Catering** - Green: ≥50%. Amber: < 30%. Red < 20%.
Victoria; 2016	Healthy Choices: Food and Drink Classification Guide	TL	**Food Outlets** and **Vending -** Green: ≥50%, Red: < 20%.	**Catering** - Green: Majority, Red: Nil. **Fundraising** - Red: Nil for children and youth. **Advertising** - Green: Majority, Red: Nil.

*NSW* New South Wales, *QLD* Queensland, *SA* South Australia, *WA* Western Australia, *TL* traffic light, *HSR* Health Star Rating

The percentages of different foods/drinks permitted in food outlets/canteens and vending, and for catering, fundraising and advertising, varied between jurisdictions and between types of institutions. In food outlets/canteens and vending, policies specified frequencies of specific foods permitted, including minimum proportions of: ‘green’ or ‘green and amber’ traffic-light classified foods/drinks, or ‘everyday’ foods; and/or maximum proportions of ‘red’ and ‘amber’ traffic-light classified foods or ‘occasional’ foods. In New South Wales, a minimum of 75% ‘everyday’ and maximum of 25% ‘occasional’ foods/drinks were permitted across all institution types, and there was a ban on sugary drinks in schools and health facilities. In the other jurisdictions, permitted proportions ranged from no ‘red’ food/drinks in most schools, to less than 20% ‘red’ in other institutions. Proportions of ‘green’ foods ranged from 50 to 60% in these jurisdictions, however many simply specified ‘majority’ to be ‘green’ and others specified a minimum of 80% combined ‘green and amber’ foods/drinks. For catering, fundraising and advertising, policies specified targets such as majority ‘green’, only ‘green’ and no ‘red’, or only ‘everyday’. Full details are displayed in Table 2.

### Comparison of nutrient criteria

The majority of policies specified that the nutrient criteria were adapted from other jurisdictions. Most were directly based on the *Fresh Tastes @ School NSW Healthy School Canteen Strategy,* or were adaptations [139]. Other policies adopted the *National Healthy School Canteen Guidelines* [6] or applied criteria based on the national guidelines.

The number of food categories targeted varied by jurisdiction, from seven in the South Australian school policy to 45 in the New South Wales health facility policy. Despite the large number of food categories targeted in many jurisdictions, few were consistently targeted across all. Overall, only five food categories were targeted as part of nutrient criteria by every state and territory in all three institution types: meat products (e.g. crumbed and coated foods, sausages, frankfurts and saveloys), hot food items (e.g. savoury pastry, pizza, oven-baked potato products, instant noodles), cakes/sweet tarts/pastries, ice-creams and dairy desserts and sweet snacks (e.g. muesli bars, sweet biscuits).

In health facilities, eight food categories were targeted by every jurisdiction with a policy, while 15 appeared in the policy of more than one state or territory. In schools, six were targeted by every jurisdiction, while 20 food categories appeared in more than one state or territory policy. For work places, seven were targeted by every jurisdiction, while 15 appeared in more than one state or territory policy. Of these commonly targeted food categories, the majority of energy and fibre criteria were consistent between jurisdictions, however only around half of the saturated fat cut-offs and less than half of the sodium criteria were consistent. For example, sodium criteria for meat products varied across all three settings, ranging from ≤450 mg to 700 mg/100 g. There were also discrepancies in saturated fat criteria across a range of food categories, with some jurisdictions applying a grams per serve cut-off and others specifying the same cut-off (g) per 100 g. A detailed summary of foods with nutrient criteria in all jurisdictions is displayed in Table 3.

**Table 3:** Summary of amber nutrient criteria for commonly targeted food categories in Australian institutions

**Food**	**Energy**	**Saturated Fat**	**Sodium**	**Sugar**	**Fibre**
**Health Facilities (ACT, NT, QLD, SA, VIC, WA)**^**a**^					
Cakes/sweet tarts/pastries	≤900 kJ/serve	≤3 g/serve (≤3/100 g SA, NT)	≤300 mg/serve (WA only)	–	≥1.5 g/serve
Savoury snacks (e.g. savoury biscuits, crisps)	≤600 kJ/serve (and some states ≤1800 kJ/100 g)	≤2-3 g/serve (≤ 3 g/100 g SA, NT)	≤200 mg/serve	–	–
Processed meat (cold luncheon and cured meats)	≤900-1000 kJ/100 g	≤3 g/100 g	≤750 mg/100 g (ACT, VIC, WA only)	–	–
Meat products (e.g. crumbed and coated meat, frankfurts)	≤1000 kJ/100 g	≤5 g/100 g	≤450-700 mg/100 g	–	–
Hot food items (e.g. savoury pastries, pizza, instant noodles)	≤1000 kJ/100 g	≤5 g/100 g	≤400 mg/100 g	–	–
Pre-prepared meals	≤2500 kJ/serve	≤2 g/100 g (≤2 g/serve VIC)	≤900 mg/serve + ≤300 mg/100 g	–	≥3 g/serve
Ice-creams and dairy desserts	≤600 kJ/serve	≤3 g/serve (≤3 g/100 g NT)	–	–	–
Sweet snacks (e.g. muesli bars, sweet biscuits).	≤600 kJ/serve	≤3 g/100 g (≤3 g/serve VIC)	–	–	≥1 g/serve
**Schools (ACT, NT, QLD, SA, TAS, VIC, WA)**^**a**^					
Meat products (e.g. crumbed and coated foods, sausages, frankfurts)	≤1000 kJ/100 g	≤5 g/100 g	≤450-700 mg/ 100 g	–	–
Hot food items (e.g. savoury pastry, pizza, oven-baked potato products, instant noodles)	≤1000 kJ/100 g	≤5 g/100 g	≤400 mg/100 g	–	–
Cakes/sweet tarts/pastries	≤900 kJ/serve	≤3 g/serve (≤3 g/100 g WA)	–	–	≥1.5 g/serve
Savoury snacks (e.g. savoury biscuits, crisps)	≤600 kJ/serve (and some states ≤1800 kJ/100 g)	≤2 g/serve	≤200 mg/serve	–	–
Ice-creams and dairy desserts	≤600 kJ/serve	≤3 g/serve	–	–	–
Sweet snacks (e.g. muesli bars, sweet biscuits)	≤600 kJ/serve	≤3 g/serve	–	–	≥1 g/serve
**Workplaces (NT, TAS, VIC, WA)**^**a**^					
Cakes/sweet tarts/pastries	≤600-900 kJ/serve	≤3 g/100 g (≤3 g/serve VIC, WA)	≤200 mg/serve (TAS only)	5-15 g/100 g (WA only)	≥1.5 g/serve
Processed meat (cold luncheon and cured meats)	≤900-1000 kJ/ 100 g (≤600 kJ/ serve WA)	≤3 g/100 g	≤700-750 mg/100 g	–	–
Meat products (e.g. crumbed and coated meat, frankfurts)	≤1000 kJ/100 g	≤5 g/100 g	≤450-700 mg/ 100 g	–	–
Hot food items (e.g. savoury pastries, pizza, instant noodles)	≤1000 kJ/100 g	≤5 g/100 g	≤400 mg/100 g	–	–
Pre-prepared meals	≤2500 kJ/serve (nil WA)	≤2 g/100 g VIC, ≤2 g/serve NT, ≤3 g/serve WA, ≤4 g/100 g TAS	≤900 mg/serve and ≤ 300 mg/ 100 g (400 mg/ 100 g WA)	–	≥3 g/serve
Ice-creams and dairy desserts	≤600 kJ/serve	≤3 g/100 g (≤3 g/serve VIC, WA)	–	5-15 g/100 g (WA only)	–
Sweet snacks (e.g. muesli bars, sweet biscuits).	≤600 kJ/serve	≤3 g/100 g (≤3 g/serve VIC, WA)	–	5-15 g/100 g (WA only)	≥1 g/serve (≥3 g/serve WA)

^a^New South Wales policies were excluded since criteria are based on portion size and Health Star Rating, rather than traffic light labelling. *ACT* Australian Capital Territory, *NT* Northern Territory, *QLD* Queensland, *SA* South Australia, *TAS* Tasmania, *VIC* Victoria, *WA* Western Australia

## Discussion

The large number of state and territory level institutional nutrition policies and guidelines in Australia highlights the perceived importance of these across the nation. This study found that all eight Australian jurisdictions have school and workplace nutrition policies/guidelines, seven have a health facility policy, and a further four policies were identified for other settings. Yet, there were notable differences in policy design and components, between institution types and jurisdictions, which may affect policy implementation, and potential impact. As an example, three-quarters of policies included implementation guides or tools, however less than half outlined procedures for monitoring and evaluation, which is a key component of any well-designed policy [140, 141]. In addition, important differences in nutrient criteria were revealed that may also be limiting policy impact. Inconsistencies between jurisdictions and institution types reveal a potential challenge for manufacturers attempting to reformulate in line with multiple different policies, and illustrates the scope for aligning and strengthening nutrition policies.

Most health facility and school nutrition policies in Australia are mandatory for government institutions and include nutrition standards. There is existing evidence that these policies can have a positive impact on dietary intake [11], though to be successful, policies must be well designed, include a number of key components, and have systems in place to support implementation, monitoring and evaluation [13]. Poor compliance in implementing policies in schools and health facilities [20, 28] has previously been documented as a major concern and has likely hindered the potential impact in Australia. A number of reasons for this have been proposed, some of which include: complexity of the product categories, concerns about loss of profit [142], lack of organisational leadership [143], lack of assistance with implementation [144], and difficulty sourcing appropriate foods [27]. Other reasons from global literature include: ambiguity in policy wording, inadequate human and financial resources, a lack of accountability mechanisms for effective monitoring and policy enforcement, and conflicts of interest between institutions and private partnerships [145]. This global evidence suggests gaps in the policy design during planning processes may also present a barrier to implementation. Our study illustrated that the majority of Australian policies contained components which are key to success, including resources to support implementation and defined roles and responsibilities [140, 141]. Yet, gaps in accountability mechanisms, such as a lack of timelines and tools for monitoring and evaluation in many jurisdictions, highlight a crucial area for improvement and suggest another reason for the poor compliance.

In contrast to the health and school policies, workplace nutrition policies are all voluntary. More importantly, only half have voluntary nutrient standards included, a gap that makes the policies difficult to operationalise. Almost all policies encouraged workplaces to develop their own guidelines and strategies based on health promoting principles by providing a policy template to modify, as opposed to adopting a state/territory-wide policy (e.g. [100, 123]). With the exception of Victoria, all workplace policies targeted a variety of non-communicable disease risk factors, such as physical activity and smoke-free environments, in addition to healthy eating. Further, the healthy eating components tended to focus on generic healthy eating strategies, such as replacing biscuits with fresh fruit [100], or displaying posters with health promotion messages [123]. The flexible policy design may incentivise workplaces to create a policy with healthy eating components, however the lack of structure and set guidance for food procurement, preparation and provision likely lessens any potential impact. Positively, many policies did include tools or resources for monitoring and evaluation, however roles and responsibilities were less likely to be defined, possibly due to the large variation in workplace sizes and structures. Similar to other institution types, the absence of this accountability mechanism may also be a barrier to implementation and monitoring of these policies.

The identification of policies in other settings illustrates large potential for future work nationwide. The Australian Food-EPI reports [29] highlight establishing a whole-of-government policy with nutrition standards to be a key next step in this area. A whole-of-government approach would apply to all government departments, and settings under government control [146]. So far, the Australian Capital Territory is the only jurisdiction with a policy for the public sector, though still retains individual policies for schools, workplaces and health facilities. However, work towards a public sector policy is underway in Victoria, and being planned in Western Australia [29]. In addition, Victoria has two unique policies targeting retailers in parks and sport/recreation centres that are under government control. These policies are relevant to all Australian jurisdictions and could be adopted or adapted for use within each jurisdiction, creating healthy food environments across a wider range of settings [7]. Further, developing a whole-of-government approach, applying across all public institutions and public settings, could eliminate the inconsistencies between policies for different institution types.

The design of institutional nutrition policies varies globally [17], depending on factors such as governance, food systems, and methods of providing and selling foods (e.g. food outlets, vending, catering and fundraising). A framework for monitoring and evaluating nutrition standards in publicly-funded institutions, based on a review of known strategies, highlights the variability in policy design [13]. In countries such as Australia and Canada, institutional nutrition policies are the responsibility of states/provinces and territories [14, 147], while in other countries these may be implemented at the national level (e.g. England [148]), or city/municipal/local government level (e.g. New York City [149]). While Australian policies are ‘nutrient-based’, other policies such as in schools in the UK are food group-based [17]. A concern with the Australian approach is that nutrient-based standards may simply lead to replacement of foods with similar foods which are lower in adverse nutrients (e.g. use sweetener instead of sugar) but still of poor nutritional value, as seen in California [150]. A combination of food and nutrient-based standards is recommended to prevent this issue occurring [13], and including food-based standards in the Australian policies could increase their impact on dietary intake. In terms of application of the policies, most Australian jurisdictions use an ‘in/out’ approach based on the nutrient criteria, in combination with categories to ‘choose most/least’. However, the New South Wales policy uses the HSR [5], a composite score approach, in combination with portion size strategies. Other countries also apply one or more of these strategies in their institutional nutrition policies, for example in schools, Bahrain uses an ‘in/out’ approach, Malaysia uses a ‘choose most/least’ approach, and Mexico uses a combination of the two [151]. Another area of difference is that Australian school policies apply to foods provided and sold, but do not include standards for the composition of meals, as seen in some policies, for example in schools in Sweden [17, 151], as Australian schools do not provide lunch meals for students. Using L’Abbé et al.’s monitoring and evaluation framework [13] to compare policies globally could provide useful insights into strengthening current institutional policies, but is outside the scope of the current paper.

The complex nature of the nutrient criteria in institutional nutrition policies, where they exist, has been previously identified as a barrier to implementation [142], and variations in criteria between jurisdictions may also be a hindrance [152]. The criteria are complex in that multiple foods and food categories are clustered together, and the wide variety of products within each group may result in challenges for institutions in procuring, preparing or providing foods meeting these criteria. For example, all meat products and alternatives are grouped together in most jurisdictions, meaning the same nutrient criteria are applied to a chicken nuggets and sausages. Yet, there are known differences in the nutrient composition of these products (e.g. sodium content [153, 154]), and individually set targets may be more appropriate. The variations between criteria for similar categories between states may also be a barrier [152], particularly as many manufacturers tend to supply food nationally, rather than within a particular jurisdiction, and therefore must manufacture products to meet the most stringent jurisdiction’s nutrient criteria. The challenges associated with the design of the institutional nutrition policies can be contrasted with the design of the national nutrient reformulation targets proposed by the Healthy Food Partnership [153]. The nutrient criteria within the proposed reformulation guideline apply to individual food categories allowing targets to be set in a format that is likely easier to understand and implement. These proposed targets will also apply nationally, eliminating any variability in criteria between states and territories, and revealing a potential way forwards for the institutional policies. Developing national institutional nutrition policies would require strong federal government leadership, supported by the state and territory governments, as the roles and responsibilities of the states and territories, and differences in health and education systems between jurisdictions [14], would require implementation to remain at the jurisdictional level.

This is the first study to investigate Australian state and territory nutrition policies across a variety of institutions. It focussed on government-led policies, as governments should be setting an example for creating healthy eating environments in publicly-funded institutions [7, 8]. The study highlighted discrepancies between jurisdictions regarding policy strength and comprehensiveness in identifying: policy gaps and critical areas for future work within schools, health facilities and workplaces, variations in the stringency of nutrition standards and nutrient criteria, and unique settings where policies could be implemented across all jurisdictions. Addressing these policy gaps and variations and expanding nutrition policies to cover a wider range of public settings would increase policy strength and comprehensiveness and likely impact on dietary intake and health. However, this study did not capture private-sector policies, or policies at local or federal level. The policies assessed were only relevant to free-living individuals, and did not capture policies relating to institutionalised individuals, such as hospital in-patients or prisoners (e.g. [155]). Policies were obtained through a manual search of state/territory health and education sector websites, supplemented by information from the Food Policy Index Australia website, and it is possible that some policies were missed. Lastly, this study only investigated differences in policy design, and did not assess the extent of implementation, barriers and enablers to implementation, or the impact of the policies on foods available, dietary intake or health outcomes. To date, much of the evaluative research has been led by the state/territory governments and data has been self-reported by institutions (e.g. [156, 157]) with minimal independent analyses or peer-review literature [20, 27]. High levels of non-compliance have also been previously highlighted [20, 28], suggesting policies are not achieving their desired impact. As such there is a need for rigorous independent evaluation of these policies to determine the current status of policy implementation, identify strategies to improve policy uptake and compliance and determine policy impact on dietary intake and health outcomes.

## Conclusions

Almost all states/territories in Australia have developed their own nutrition policies for schools, workplaces and health facilities, though policies vary between jurisdictions and institution types. This study highlights gaps in existing policies, such as a lack of monitoring and evaluation tools and timelines, which may be a barrier to implementation and prevent the policies having their intended impact. Rigorous independent evaluation of these policies is needed to identify strategies to improve uptake and compliance.

## Data Availability

The datasets generated and analysed during the current study are publicly available from health and education department websites for each Australian state and territory government, from the Food Policy Index Australia website or other stakeholder websites, as referenced in the text.

## Abbreviations

ACT: Australian Capital Territory
NSW: New South Wales
NT: Northern Territory
QLD: Queensland
SA: South Australia
TAS: Tasmania
VIC: Victoria
WA: Western Australia
TL: Traffic light
HSR: Health Star Rating
